# The impact of centralized coronary stent procurement program on acute myocardial infarction treatments: evidence from China

**DOI:** 10.3389/fpubh.2023.1285558

**Published:** 2023-11-30

**Authors:** Weiyan Jian, Wuping Zhou, Lanyue Zhang

**Affiliations:** Department of Health Policy and Management, School of Public Health, Peking University, Beijing, China

**Keywords:** centralized procurement, coronary stents, acute myocardial infarction, treatment behavior, China

## Abstract

**Background:**

The advent of coronary stents has resulted in many more many lives being saved from acute myocardial infarction (AMI). However, the high price associated with this method of treatment also imposes a heavy economic burden on healthcare systems. As a country making significant use of coronary stents, in 2021, China introduced a program around this method of treatment grounded in centralized procurement and it is the focus of this paper to assess the impact of this policy on AMI treatments.

**Methods:**

The patients with AMI are selected as the study group, and the patients with pre-cerebral vascular stenosis are selected as the control group, and individual-level medical insurance settlement data are collected from the years 2018, 2019, and 2021. Differences-in-differences methodology is used to analyze the impacts of this program on the probability changes in respect of AMI patients receiving stent therapy, as well as changes relating to cost, length of stay and 30-day readmission.

**Results:**

The results show that the reform has led to a reduction in the probability of AMI patients using stents to 51% of the original rate. Additionally, the average cost is shown to have decreased by 41%, and no significant changes can be found in respect of the length of stay and 30-day readmission.

**Conclusion:**

In sum, the centralized procurement program is shown to reduce not only the medical expenses incurred by treating patients with AMI, but also the use of coronary stents, resulting in changes to the treatment patterns of patients with AMI.

## 1 Introduction

Although acute myocardial infarction (AMI) has historically been associated with a high risk of death, the invention of interventional treatment techniques has resulted in the effective reduction of the mortality rate of patients with this disease (1, 2). Coronary stents are the core medical consumables in interventional treatment, and percutaneous coronary interventions (PCI) with stents has become a routine form of treatment for AMI patients (with PCI with balloon being another surgical method used to treat AMI, in addition to traditional coronary bypass surgery) (3, 4). However, coronary stents are expensive to produce and so increases in their usage has given rise to considerable pressures operating on healthcare systems.

The practice of centralized procurement, also known as volume-based procurement, has come to occupy a central position as a policy instrument for reducing public spending through increasing the market power of the payer (5–9). The World Health Organization recommends the use of this method to low- and middle-income countries, and many countries have benefited its introduction (10). For example, Greece has managed to secure substantial expenditure reductions through public procurement and the joint purchasing of medical devices and products (11). Central procurement practices have also been successful in relation to securing optimum prices in South Africa and Brazil. And in Italy, through the introduction of regional Central Purchasing Bodies (CPB), the local health service providers have achieved a 2–8% reduction in health expenditure, including reduced expenditure on health-related goods such as surgical devices and on health services more generally (12–14).

In China, the coverage rate of social health insurance has reached 98%, and, as the single payer, China’s social health insurance has the bargaining power necessary to engage in centralized procurement in a successful manner. China introduced a centralized procurement program for expensive drugs in 2019 (15, 16). Following this, the centralized procurement program was extended to coronary stents in 2021.

Rates of use of coronary stents in China are considerable. Between 2009 and 2018, the use of coronary stents increased dramatically from 230 thousand to more than 1.5 million, at a cost of 15 billion RMB (2.2 billion USD) (17). Under the centralized procurement program, the market share of the winning coronary stent in pilot cities is often more than 70%. This has been accompanied by a consequent sharp drop in price: whereas the average selling price of coronary stents was around 13,000 RMB (nearly 2,000 USD) before centralized procurement, it has now dropped to around 700 RMB (around 110 USD) (17).

The question of whether the centralized procurement of coronary stents has the potential to reduce medical expenses incurred in the treatment of AMI patients is one that must be answered through consideration of real-world data. Moreover, existing evidence shows that the procurement policy has an impact not only on medical expenses. For example, following the establishment of a centralized bidding procurement system in Shandong, antibiotic consumption in that region significantly decreased (18, 19). The reason for this is likely that antibiotics were previously overused and the advent of centralized procurement drove down the price of antibiotics in a way that resulted in the significant reduction of profits from sales, thus removing providers’ incentive to induce demand (20). Against this background, this paper assesses the impact of the coronary stent policy on the medical expenses of AMI patients while also analyzing changes in providers’ treatment patterns following the reform and the resulting changes in the quality of healthcare provision.

## 2 Materials and methods

### 2.1 Data collection

A city in inland China with a population of 2 million (City Z) was chosen as the sample area. This city had previously been selected as one of the pilot cities for the centralized procurement policy in respect of coronary stents. In terms of comparisons with other large cities in inland China, the *per capita* GDP and *per capita* medical resources are above average, although the population structure is representative of such cities. The medical insurance coverage rate in City Z is 98%, and the out-of-pocket payment rate for hospitalized patients is around 30%. This is the general level in large mainland Chinese cities.

The study group for this project was set to include all instances of AMI in 2018, 2019, and 2021 from the claim dataset of patients covered by social medical insurance in City Z. Variables included each patient’s primary diagnosis, surgical procedures, medical costs, hospital admission and discharge times, and basic information such as age and sex. Cases with a primary diagnosis marked by ICD-10 code I21.0-I21.9 (AMI) were included as part of the dataset. Surgical procedures used ICD-9-CM3, i.e., PCI with stents (ICD-9-CM3 36.06 and 36.07). Because inpatient services were affected by the COVID-19 pandemic in the first half of 2020, the data for this year were excluded (21). Entering 2021, the prevalence of COVID-19 in City Z can be observed to have been low (the number of new cases in each month of the year not exceeding 100), and so it can be concluded that the impact of COVID-19 on local healthcare accessibility was not significant.

The control group for this study was set to comprise cases of pre-cerebral vascular stenosis (PCVS, ICD code I63.2 and I65.X). The reason for selecting these cases as the control group was that there is a certain probability of using cerebral vascular stents (non-coronary stents) in the treatment of these cases, while the centralized procurement in China this time included only the use of coronary stents, not cerebral vascular stents. In other words, the treatment of cases with cerebral vascular stenosis would not have been affected by the centralized procurement policy. Moreover, among the various types of stents procured in City Z, the majority are coronary stents, followed by cerebral vascular stents.

### 2.2 Research hypotheses and analysis indicators

The primary hypothesis was that the expenditure on AMI patients who received PCI with stents would be seen to reduce significantly following the implementation of centralized procurement. Additionally, it was also hypothesized that if it was found that there was overuse of coronary stents originally, then that the proportion of AMI patients who had received PCI with stents would decrease as a result of the centralized procurement policy reducing the profit margins associated with selling coronary stents.

Given these hypotheses, this study measures the medical expenditure of AMI patients and the probability of the occurrence of PCI with stents. Length of stay is the efficiency indicator and 30-day readmission is taken to be the indicator of quality of care.

### 2.3 Statistical methods

A difference-in-differences (DID) model is used to test the underlying hypotheses stated above. AMI cases are set as the treatment group, while PCVS cases are set as the control group. Starting from the DID approach, 2 years of data from before the procurement reform (2018 and 2019) are first used to conduct a parallel trend test. If the parallel trend test is passed, then there is an evaluation of the effect of centralized procurement using data from 2019 (representing the situation before the implementation of the centralized procurement program) and from 2021 (reflecting the situation after the introduction of the centralized procurement program). This is done in the form specified in Eq. (1):


(1) $$\begin{array}{l} Y=\beta_{0}+\beta_{1}*AMI+\beta_{2021}{\mathrm{Year}}_{2021}\\ \;+\delta *{\mathrm{Year}}_{2021}*AMI+\sum \beta_{i}*X_{i}+\varepsilon \end{array}$$

in which the variable Y represents the probability of patients receiving stent treatments as well as other outcome measures, such as expenditure, length of stay and 30-day readmission. The variable AMI is an indicator that identifies whether the cases are cases of AMI. ${\mathrm{Year}}_{2021}$ is a dummy variable indicating whether the observation is from 2021. $X_{i}$ represents the control variables included in the analysis. We chose Sex, Age and Charlson Index as covariates. The inclusion of the patient’s sex as a covariate is based on the well-established differences in presentation, diagnosis, and treatment outcomes for AMI, between males and females (22, 23). Age is a crucial determinant in cardiovascular health. Furthermore, age-related comorbidities and the physiological changes that come with aging can influence the outcomes we are examining (24, 25). And Charlson Index (CCI) is a widely recognized tool for assessing the risk of mortality from comorbidities (26). Including the Charlson Index in our model allows us to adjust for the confounding effects of comorbidities, ensuring that our findings reflect the impact of the intervention itself and not the underlying health status of the patients. When Y represents the probability of patients receiving stent treatments, the 30-day readmission rate, a linear probability model is employed. When Y represents expenditure or length of stay, the logarithm of these variables is taken, and a linear regression model is used. Standard errors were clustered at the hospital level to account for potential correlation within hospitals. We’ve included robustness tests in Supplementary material, to confirm the consistent trend in our main results (details are provided in Supplementary Table 1). The statistical analysis was conducted using Stata Version 17.0.

### 2.4 Ethics approval statement

The studies involving humans were approved by Peking University (IRB number: IRB00001052–18005). The studies were conducted in accordance with the local legislation and institutional requirements. Written informed consent for participation was not required from the participants or the participants’ legal guardians/next of kin in accordance with the national legislation and institutional requirements.

## 3 Results

Table 1 presents the sample data characteristics. For this study, a total of 4,069 cases with AMI and 406 cases with PCVS were screened from 2019 and 2021. Most of the discharged patients were male, and the distribution of sex has no statistical difference across different years. Irrespective of whether the cases in question are AMI or PCVS cases, those affecting persons over 60 years old account for more than 60%; in terms of comorbidities, patients without comorbidities make up a higher proportion, and the proportion of cases with serious comorbidities (CCI > =2) in 2021 is higher than in 2019.

**Table 1:** The sample data characteristics of AMI patients.

Factor	AMI			PCVS		
Year	2019	2021	*p*-value	2019	2021	*p*-value
Number of cases	1945	2,124		159	247	
Sex (N, %)						
Male	1,411 (72.5)	1,501 (70.7)		114 (71.7)	181 (73.3)	
Female	534 (27.5)	623 (29.3)	0.19	45 (28.3)	66 (26.7)	0.73
Age (*N*, %)						
19–40	31 (1.6)	63 (3.0)	<0.001	3 (1.9)	3 (1.2)	0.049
40–60	581 (29.9)	749 (35.3)		32 (20.1)	74 (30.0)	
60–80	1,003 (51.6)	1,079 (50.8)		99 (62.3)	148 (59.9)	
>80	330 (17.0)	233 (11.0)		25 (15.7)	22 (8.9)	
Charlson index						
0	1,000 (51.4)	638 (30.0)	<0.001	101 (63.5)	144 (58.3)	<0.001
1	461 (23.7)	670 (31.5)		36 (22.6)	38 (15.4)	
2	311 (16.0)	421 (19.8)		16 (10.1)	41 (16.6)	
> = 3	173 (8.9)	395 (18.6)		6 (3.8)	24 (9.7)	

AMI, acute myocardial infarction; PCVS, pre-cerebral vascular stenosis.

Table 2 shows the changes in outcome indicators for the intervention groups and control group before and after centralized procurement. Among the AMI cases, the proportion of cases receiving stent treatment can be seen to initially decrease from 56.3% in 2019 to 51.5% following the reform. However, among PCVS patients, the proportion of cases receiving stent treatment can be seen to increase from 13.8% in 2019 to 19.4% in 2021, indicating that the research group and the control group demonstrate opposite trends. The same situation also appears in respect of the average cost. After adjusting the expenditure associated with using the Consumer Price Index (CPI), there remains a significant reduction in average expenditure per case in terms of AMI patients, from 38,503 RMB to 27,333 RMB in 2021. In relation to PCVS cases, medical expenses can be seen to have increased by 2931RMB. As for the length of hospital stay, both the research group and the control group demonstrate a downward trend, with a slightly higher decrease in the research group. And regarding the 30-day readmission rate, the research group shows an increase from 2.0 to 3.3%, while the control group remains relatively stable.

**Table 2:** Descriptive analysis of outcome indicators for the intervention group and control group before and after centralized procurement.

Outcome	AMI			PCVS			
Year	2019	2021	Diff (%)	2019	2021	Diff (%)	DID (%)
PCI with stent (*N*, %)	1,095 (56.3)	1,085 (51.1)	−5.2	22 (13.8)	48 (19.4)	5.6	−10.8
PCI with balloon (*N*, %)	42(2.2)	129 (6.1)	3.9	——	——	——	——
CABG (*N*, %)	9 (0.5)	15 (0.7)	0.2	——	——	——	——
Average medical expenditure (mean, SD)	38,503 (32970)	27,333 (23670)	−9,300	26,049 (34230)	28,981 (33017)	−1212.9	−8087.2
Average length of stay (mean, SD)	8.9 (7.5)	8.0 (5.2)	−10.0	11.5 (8.1)	10.6 (8.1)	−8.2	−1.8
30-day readmission rate (*N*, %)	39 (2.0)	71 (3.3)	1.3	3 (1.9)	5 (2.0)	0.1	1.2

AMI, acute myocardial infarction; PCVS, pre-cerebral vascular stenosis; PCI, percutaneous coronary interventions; CABG, coronary artery bypass grafting; SD: standard deviation; DID, difference-in-differences.

Another notable finding is that, in respect of AMI cases, while the probability of receiving PCI with stent decreases, the probability of receiving PCI with balloon and coronary artery bypass grafting (CABG) both increase. This is especially evident in respect of the former, which can be seen to have nearly doubled.

After verifying that the research group and control group did indeed present the same trend for each outcome indicator before centralized procurement (details are provided in Supplementary Table 2), regression analysis based on the DID methodology was used to assess the impact of centralized procurement. Table 3 shows the results of the regression analysis. It can be seen that, following the introduction of centralized procurement, the probability of AMI patients receiving stent treatment decreased by 11% and the average medical cost decreased by 41%, with statistically insignificant changes in hospitalization time and 30-day readmission rates. In addition, the retrospective model also shows that the probability of PCI with stent and the medical costs are both higher; older adult patients (> = 81 years old) and patients with more serious comorbidities (CCI > 2) have a lower probability of receiving PCI with stent.

**Table 3:** Regression analysis results of the impact of centralized procurement program policy.

	Probability of receiving stent treatments (Linear Probability)		Expenditure (Log-linear)		Length of stay (Log-linear)		30-day readmission (Linear Probability)	
	β	95% CI	β	95% CI	β	95% CI	β	95% CI
Effect of policy	−0.11^**^	(−0.22–−0.00)	−0.41^**^	(−0.75–−0.08)	−0.02	(−0.23–0.18)	0.01	(−0.01–0.02)
AMI	0.44^***^	(0.34–0.54)	0.55^***^	(0.22–0.88)	−0.28^***^	(−0.44–−0.12)	0.00	(−0.01–0.01)
Year = 2021	0.08	(−0.03–0.18)	0.15	(−0.19–0.49)	−0.13	(−0.35–0.09)	0.01^*^	(−0.00–0.02)
Sex (Ref)								
Male	0.10^***^	(0.08–0.12)	0.16^***^	(0.09–0.23)	0.01	(−0.02–0.05)	0.00	(−0.00–0.01)
Age (Ref)								
41–60	0.07^**^	(0.01–0.12)	0.12^*^	(−0.02–0.25)	0.05	(−0.11–0.21)	0.01^***^	(0.01–0.02)
61–80	0.00	(−0.05–0.04)	0.04	(−0.08–0.16)	0.03	(−0.11–0.17)	0.02^***^	(0.01–0.03)
> = 81	−0.20^***^	(−0.27–−0.12)	−0.30^***^	(−0.44–−0.15)	−0.02	(−0.15–0.11)	0.03^***^	(0.01–0.04)
CCI (CCI = 0 Ref)								
=1	−0.06	(−0.14–0.02)	−0.10	(−0.32–0.12)	0.06	(−0.04–0.17)	−0.02^***^	(−0.03–−0.01)
=2	−0.15^*^	(−0.31–0.00)	−0.17	(−0.48–0.14)	0.09^**^	(0.00–0.18)	−0.01	(−0.03–0.02)
> = 3	−0.22^**^	(−0.40–−0.04)	−0.07	(−0.37–0.22)	0.15^***^	(0.08–0.22)	−0.01	(−0.04–0.03)

AMI, acute myocardial infarction.

*** *p* < 0.01.

** *p* < 0.05.

* *p* < 0.1.

## 4 Discussion

This paper evaluates the impact of the centralized procurement of coronary stents on the treatment of AMI cases. The results reported above show that the centralized procurement policy used in City Z is associated with a significant reduction in the medical costs of AMI patients and a decrease in the probability of AMI patients receiving PCI with stents. The reduction in medical costs for AMI patients is to be expected given the widespread trend of centralized procurement resulting in the reduction of coronary stent costs. The decrease in AMI cases receiving PCI with stents is likely due to the overuse of coronary stents prior to the reform (27). Centralized procurement has significantly reduced the profits from selling coronary stents, and the influence of manufacturers on providers has also decreased as a result (28); therefore, providers’ behavior of inducing patients to use coronary stents may also have decreased (29).

There is an indirect corroboration of the speculation that there was overuse of coronary stents before centralized procurement, and this is reflected in the fact that AMI cases that received another type of PCI (PCI with balloon) increased with the centralized procurement of coronary stents. Before centralized procurement, the price of balloons was much lower than stents, and balloons were not included in this centralized procurement policy. After the price of coronary stents was reduced through centralized procurement, the use of balloons increased in a “substitutive” manner; the providers of medical insurance would have noticed the issue of the increasing use of balloons and thus included balloons in the centralized procurement policy in 2022 (30).

Previous studies of centralized procurement have mainly focused on how medical costs can be reduced by directly lowering the acquisition prices of medical devices or drugs with only a few considering the impact centralized procurement on the behaviors of healthcare providers (29, 31, 32).

Centralized procurement has the potential to have a considerable impact on manufacturers’ profits, and their strong opposition may cause difficulties in the implementation of the procurement policy. For example, the Inflation Reduction Action in the USA allows Medicare to negotiate prices for selected drugs, potentially saving significant funds. Nevertheless, this initiative has encountered vehement resistance from major pharmaceutical corporations. Beyond threatening Medicare with litigation, such corporations have also expressed concerns that centralized procurement could curtail their investments in the research and development of novel treatments (33). In New Zealand, some manufacturers who previously won bids were unable to provide sufficient quantities of drugs at the contract price of centralized procurement (34).

Another concern relates to whether changes in the behavior of healthcare providers following the implementation of centralized procurement will affect the quality of care. In their study on a sample of Italian hospitals, Ferraresi et al. (35) showed that the quality of care was not affected by the introduction of a centralized procurement program, pointing to exact measures of the mortality and discharge rates associated with seven diseases, including AIDS and diabetes (35). The regression results of this study show no significant increase in the 30-day readmission rate for AMI patients following the centralized procurement of coronary stents. However, the long-term impacts on quality remain to be seen, especially given that the descriptive results show a trend of increased 30-day readmission following the introduction of centralized procurement.

In terms of the limitations of this study, first, it has focused only on the short-term effects of centralized programs in relation to high-value medical consumables, and so longer-term evaluation remains to be done. Secondly, due to data limitations, it was not possible to analyze the changes in numbers of cases of PCI with stents.

## 5 Conclusion

The centralized coronary stent procurement program introduced in China has significantly reduced the medical expenditure associated with AMI patients in this country. This program has also resulted in greater control over the overuse of coronary stents. Given these facts, the centralized procurement policy is likely to continue to be favored by medical insurance providers. However, for the policy to continue developing positively, it is necessary, first, to resist opposition from manufacturers and to perhaps even introduce supporting policies to maintain enthusiasm for technological innovation by manufacturers; and second, there is a need for continuous monitoring of the impact of centralized procurement on the quality of medical products.

## Data availability statement

The datasets presented in this article are not readily available because they cannot be shared publicly as consent was not obtained. Requests to access the datasets should be directed to WJ, jianwy@126.com.

## Ethics statement

The studies involving humans were approved by Peking University (IRB number: IRB00001052–18005). The studies were conducted in accordance with the local legislation and institutional requirements. Written informed consent for participation was not required from the participants or the participants’ legal guardians/next of kin in accordance with the national legislation and institutional requirements.

## Author contributions

WJ: Conceptualization, Methodology, Project administration, Resources, Supervision, Writing – original draft, Writing – review & editing. WZ: Data curation, Formal analysis, Writing – review & editing, Methodology. LZ: Writing – original draft, Writing – review & editing, Methodology.
