# Supplementary material for: The impact of centralized coronary stent procurement program on acute myocardial infarction treatments: evidence from China
Source: Front Public Health. 2023 Nov 30;11:1285558. doi: 10.3389/fpubh.2023.1285558 (PMC10720903; doi:10.3389/fpubh.2023.1285558)
Supplement: Supplementary file 1 [file Table_1.DOCX]

Supplementary Material

# Supplementary Table 1. Robustness Test Results.

We conducted robustness checks using two methods to ensure that the regression results were not due to chance, but a consequence of policy intervention.

Firstly, we pooled the data from 2018 and 2019 together to construct a new control group, thereby observing the impact of the policy over a longer period. We could see that, even with a larger sample size and a longer time span, there was a significant reduction in stent usage and costs in 2021 when using the expanded control group. Although the inclusion of additional years in the control group introduced potential and unknown confounding factors, these disturbances were much smaller than the effects brought about by the centralized procurement policy, as could be clearly seen from the changes in the coefficients. Thus, we can infer that the policy changes in 2021 indeed had an important and robust impact.

Secondly, we used logistic regression to analyze the probability of AMI patients using stents and the 30-day readmission rate, and Linear-Linear models to analyze costs and length of hospital stay, in order to avoid false positives caused by specific statistical models. The results indicate that, regardless of whether we used logistic models or linear probability models, or Log-Linear models or Linear-Linear models, we observed a significant decrease in the probability of stent usage and medical expenses. This significance is not introduced by the inference methods of the models but is objectively present.

Based on the robustness checks mentioned, we can observe that the trends are consistent with the results of the models used in the main text. This consistency across different analytical approaches lends further credence to our findings, reinforcing the validity of our conclusions drawn from the primary analysis.

# Robustness Test Result 1: Using 2018 and 2019 as the Control Group.

|  | Probability of receiving stent treatments  (Linear Probability) | | Expenditure  (Log-linear) | | Length of stay  (Log-linear) | | 30-day readmission  (Linear Probability) | |
| --- | --- | --- | --- | --- | --- | --- | --- | --- |
|  | β | 95% CI | β | 95% CI | β | 95% CI | β | 95% CI |
| Effect of policy | -0.09** | (-0.18 - -0.00) | -0.41** | (-0.78 - -0.05) | -0.02 | (-0.24 - 0.21) | -0.00 | (-0.02 - 0.01) |
| AMI | 0.41*** | (0.30 - 0.52) | 0.54*** | (0.17 - 0.91) | -0.29*** | (-0.47 - -0.11) | 0.02*** | (0.01 - 0.02) |
| Year = 2021 | 0.09** | (0.01 - 0.17) | 0.20 | (-0.15 - 0.54) | -0.15 | (-0.38 - 0.09) | 0.02** | (0.00 - 0.03) |
| Sex = male | 0.11*** | (0.08 - 0.14) | 0.21*** | (0.11 - 0.30) | 0.03* | (-0.00 - 0.06) | -0.00 | (-0.01 - 0.01) |
| Age |  |  |  |  |  |  |  |  |
| 41-60 | 0.06** | (0.00 - 0.11) | 0.12* | (-0.01 - 0.25) | 0.04 | (-0.09 - 0.17) | 0.01*** | (0.01 - 0.02) |
| 61-80 | -0.00 | (-0.05 - 0.04) | 0.03 | (-0.09 - 0.16) | 0.03 | (-0.08 - 0.14) | 0.02*** | (0.02 - 0.03) |
| >=81 | -0.20*** | (-0.25 - -0.15) | -0.25*** | (-0.39 - -0.12) | 0.02 | (-0.09 - 0.13) | 0.02*** | (0.01 - 0.03) |
| CCI |  |  |  |  |  |  |  |  |
| =1 | -0.09* | (-0.19 - 0.01) | -0.20 | (-0.49 - 0.09) | 0.01 | (-0.10 - 0.13) | -0.01** | (-0.02 - -0.00) |
| =2 | -0.18* | (-0.36 - 0.00) | -0.28 | (-0.70 - 0.15) | 0.03 | (-0.08 - 0.14) | -0.00 | (-0.02 - 0.01) |
| >=3 | -0.23** | (-0.43 - -0.03) | -0.17 | (-0.52 - 0.18) | 0.11*** | (0.05 - 0.18) | -0.00 | (-0.03 - 0.02) |

# Robustness Test Result 2: Using Different Statistical Models.

|  | Probability of receiving stent treatments  (Logistics model) | | Expenditure  (Linear-linear) | | Length of stay  (Linear -linear) | | 30-day readmission  (Logistics model) | |
| --- | --- | --- | --- | --- | --- | --- | --- | --- |
|  | β | 95% CI | β | 95% CI | β | 95% CI | β | 95% CI |
| Effect of policy | 0.51** | (0.31 - 0.86) | -13,940.79*** | (-21,714.97 - -6,166.61) | 0.28 | (-2.49 - 3.04) | 1.30 | (0.79 - 2.15) |
| AMI | 9.48*** | (5.52 - 16.30) | 12,467.26*** | (5,748.08 - 19,186.45) | -2.82*** | (-4.40 - -1.23) | 1.14 | (0.68 - 1.91) |
| Year = 2021 | 1.68* | (0.92 - 3.09) | 2,294.47 | (-6,066.78 - 10,655.73) | -1.50 | (-4.78 - 1.78) | 1.50 | (0.91 - 2.49) |
| Sex = male | 1.59*** | (1.44 - 1.75) | 4,125.08*** | (2,813.94 - 5,436.22) | 0.16 | (-0.06 - 0.38) | 1.14 | (0.87 - 1.50) |
| Age |  |  |  |  |  |  |  | (1.15 - 5.37) |
| 41-60 | 1.35*** | (1.08 - 1.69) | 3,902.33** | (524.13 - 7,280.52) | 0.39 | (-0.55 - 1.33) | 2.49** | (1.09 - 10.84) |
| 61-80 | 1.01 | (0.83 - 1.22) | 3,969.31** | (704.92 - 7,233.70) | 0.71* | (-0.11 - 1.53) | 3.43** | (1.06 - 14.85) |
| >=81 | 0.41*** | (0.29 - 0.58) | -4,689.36*** | (-6,884.09 - -2,494.62) | 0.78* | (-0.04 - 1.61) | 3.97** | (0.28 - 0.76) |
| CCI |  |  |  |  |  |  |  | (0.33 - 1.78) |
| =1 | 0.77 | (0.56 - 1.07) | -296.41 | (-4,668.56 - 4,075.74) | 0.96** | (0.15 - 1.77) | 0.46*** | (0.21 - 2.46) |
| =2 | 0.51** | (0.27 - 0.96) | -84.61 | (-5,184.11 - 5,014.90) | 1.74*** | (1.09 - 2.39) | 0.77 | (0.79 - 2.15) |
| >=3 | 0.38** | (0.17 - 0.83) | 1,222.42 | (-2,687.73 - 5,132.56) | 2.29*** | (1.03 - 3.56) | 0.71 | (0.68 - 1.91) |

# Supplementary Table 2. Results of Parallel Trend Test Using Double-difference Method Based on Data From 2018 and 2019.

The purpose of the parallel trends test is to verify whether, in the absence of policy intervention, the direction and magnitude of outcome variable changes in the intervention group and the control group remain consistent, that is whether the regression coefficients are significant. When the regression coefficients are not significant, we have reason to believe that the parallel trends assumption holds, and the DID method is applicable. As shown in the table below, the results of the parallel trends test in this study are not significant.

|  | Probability of receiving stent treatments  (Linear Probability) | | Expenditure  (Log-linear) | | Length of stay  (Log-linear) | | 30-day readmission  (Linear Probability) | |
| --- | --- | --- | --- | --- | --- | --- | --- | --- |
|  | β | 95% CI | β | 95% CI | β | 95% CI | β | 95% CI |
| Parallel trend | 0.07 | (0.00 - 0.14) | 0.08 | (-0.22 - 0.37) | 0.04 | (-0.05 - 0.13) | -0.03 | (-0.04 - -0.02) |
| AMI | 0.38*** | (0.25 - 0.50) | 0.51** | (0.05 - 0.96) | -0.30*** | (-0.51 - -0.10) | 0.03*** | (0.02 - 0.04) |
| Year = 2019 | -0.01 | (-0.10 - 0.09) | -0.02 | (-0.26 - 0.22) | -0.09* | (-0.19 - 0.01) | 0.02*** | (0.01 - 0.03) |
| Sex = male | 0.13*** | (0.09 - 0.17) | 0.26*** | (0.13 - 0.38) | 0.03 | (-0.02 - 0.09) | -0.01* | (-0.02 - 0.00) |
| Age |  |  |  |  |  |  |  |  |
| 41-60 | 0.06 | (-0.05 - 0.17) | 0.02 | (-0.33 - 0.36) | -0.01 | (-0.16 - 0.14) | 0.01*** | (0.00 - 0.02) |
| 61-80 | 0.00 | (-0.10 - 0.10) | -0.09 | (-0.40 - 0.21) | -0.01 | (-0.14 - 0.12) | 0.02*** | (0.01 - 0.03) |
| >=81 | -0.20*** | (-0.26 - -0.14) | -0.37*** | (-0.60 - -0.14) | -0.01 | (-0.10 - 0.09) | 0.02** | (0.00 - 0.03) |
| CCI |  |  |  |  |  |  |  |  |
| =1 | -0.12 | (-0.27 - 0.02) | -0.33 | (-0.75 - 0.10) | -0.05 | (-0.21 - 0.12) | 0.00 | (-0.02 - 0.01) |
| =2 | -0.22** | (-0.44 - -0.01) | -0.45 | (-1.09 - 0.19) | -0.06 | (-0.27 - 0.15) | -0.01 | (-0.02 - 0.01) |
| >=3 | -0.26** | (-0.49 - -0.04) | -0.42 | (-0.92 - 0.09) | 0.08 | (-0.04 - 0.21) | 0.00 | (-0.02 - 0.01) |

*** p<0.01, ** p<0.05, * p<0.1

AMI: acute myocardial infarction
